# Crocin Mitigates Glutamate Excitotoxicity and Tau Hyperphosphorylation by Modulating EAAT2 and Akt/Tau Pathway in a Scopolamine-induced Rat Model of Alzheimer’s Disease

**DOI:** 10.1007/s11064-026-04692-z

**Published:** 2026-03-05

**Authors:** Safinaz E. El-Baga, Mohammed H. Hassan, Eatemad A. Awadalla, Abd El-Kader M. Abd El-Kader

**Affiliations:** 1 Department of Zoology, Faculty of Science, Aswan University, Aswan, 81528 Egypt; 2 Department of Medical Biochemistry and Molecular Biology, Faculty of Medicine, Qena University, 83523 Qena, Egypt

**Keywords:** Alzheimer’s disease, Crocin, Memory impairment, Excitotoxicity, EAAT2, Akt/Tau

## Abstract

Alzheimer’s disease (AD) is characterized by glutamatergic dysregulation and excitotoxicity, largely associated with impaired activity of the excitatory amino acid transporter 2 (EAAT2). Downregulation of EAAT2 results in glutamate accumulation, N-Methyl-D-Aspartate (NMDA) receptor overactivation, and neuronal injury. Crocin (Cr), a carotenoid compound extracted from saffron (*Crocus sativus*), exhibits potent antioxidant and neuroprotective properties, particularly in experimental models of neurodegeneration. Forty-eight adult male rats were divided into six groups: control (saline), crocin (50 mg/kg), scopolamine (3 mg/kg for 7 days), scopolamine followed by memantine (M) (20 mg/kg), scopolamine followed by crocin, and scopolamine followed by both memantine and crocin. This study aimed to evaluate the therapeutic potential of crocin, alone and in combination with memantine, in a scopolamine-induced rat model of Alzheimer’s disease, with a focus on EAAT2 modulation. Scopolamine administration significantly elevated glutamate, NMDAR and p-tau levels while reducing p-Akt, GABA and EAAT2 levels, accompanied by marked hippocampal neurodegeneration. In contrast, crocin treatment, either alone or in combination with memantine, restored neurotransmitter balance, downregulated NMDAR, upregulated EAAT2, increased p-Akt expression level and reduced tau phosphorylation. Histological analysis further confirmed notable structural recovery of hippocampal neurons.

**Supplementary Information:**

The online version contains supplementary material available at 10.1007/s11064-026-04692-z.

## Introduction

Alzheimer’s disease (AD) is a destructive and progressive neurodegenerative disorder with multiple proposed etiologies. It correlated with spatial disorientation, memory loss, and a decline in intellectual capacity [1, 2]. Despite decades of intensive research, the precise etiology of AD remains incompletely understood. Several interrelated hypotheses have been proposed, including cholinergic dysfunction, extracellular β-amyloid (Aβ) accumulation, tau hyperphosphorylation, glutamate excitotoxicity, oxidative stress, and neuroinflammation [3–5]. However, none of these fully explain disease progression, and to date no curative therapy exists. Thus, identifying novel therapeutic targets that address the multifactorial pathology of AD remains a major priority.

Neurotransmitters, the nervous system’s chemical messengers, are essential for maintaining the integrity of neuronal communication [6, 7]. Latest advances have mentioned the potential of exploring the function of glutamatergic systems in the pathophysiology of AD [8]. In AD, dysregulated glutamatergic transmission leads to excessive extracellular glutamate levels, contributing to excitotoxic neuronal damage [9].

Excessive glutamate overstimulates N-methyl-D-aspartate (NMDA) receptors, causing sustained calcium influx into postsynaptic neurons, mitochondrial dysfunction, oxidative stress, and ultimately neuronal death — a process termed *excitotoxicity* [10]. Under physiological conditions, excess glutamate is rapidly cleared from the synaptic cleft by astrocytic excitatory amino acid transporters (EAATs), primarily EAAT2, which accounts for approximately 90–95% of total glutamate uptake [11–18]. Importantly, reduced EAAT2 expression and activity have been consistently reported in AD patients and animal models, implicating EAAT2 dysfunction as a critical driver of disease progression [19].

Sustained glutamatergic stress can impair pro-survival signaling, particularly the phosphoinositide-3-kinase/protein kinase B (PI3K/Akt) pathway, which normally inhibits glycogen synthase kinase-3β (GSK-3β) [20, 21]. In AD, disrupted PI3K/Akt signaling leads to unchecked GSK-3β activity, promoting aberrant tau phosphorylation [22, 23]. This interplay links glutamate dysregulation to tau pathology and highlights the broader consequences of EAAT2 impairment.

Although there is no efficient cure that delays the progression of AD, numerous studies have found that using natural products as expected treatments for neurodegeneration has health-promoting properties [24, 25]. Crocin is a water-soluble carotenoid compound primarily responsible for the characteristic color of saffron (*Crocus sativus*). It has gained considerable attention for its potent antioxidant, anti-inflammatory, and neuroprotective properties [26, 27]. Recently, numerous studies have demonstrated that crocin may serve as a multifunctional therapeutic agent with neuroprotective properties, potentially offering both protective and therapeutic benefits for AD [28]. Crocin has been proved to reduce Aβ aggregation and recover learning and memory deficits in AD [29]. Its anti-amyloidogenic effects are exerted by preventing Aβ formation and neurofibrillary tangle development, disrupting amyloid aggregates, reducing β- and γ-secretase activity responsible for harmful Aβ peptides, and reducing both total tau and phosphorylated tau levels [30–32].

Currently, memantine, a non-competitive NMDA receptor antagonist, is the only approved AD drug that directly targets the glutamatergic system. By attenuating NMDA receptor overactivation, memantine partially alleviates excitotoxicity in moderate-to-severe AD [33–36]. However, its clinical efficacy is limited, underscoring the need for adjunctive or alternative approaches that target upstream glutamate clearance mechanisms.

Therefore, the present study aimed to investigate the therapeutic potential of crocin in an experimental model of AD, with a particular focus on its ability to modulate EAAT2 activity and restore glutamatergic balance. Furthermore, crocin’s effects were compared with memantine, either alone or in combination, to evaluate whether such interventions could attenuate excitotoxicity and downstream Akt/GSK-3β/p-Tau signaling abnormalities, thereby mitigating neurodegenerative processes associated with AD.

## Materials and Methods

### Materials

Scopolamine (Sc) was bought from Fluka Bio Chemika Co. USA (product no. 37022), Crocin (Cr) was obtained from Sigma Aldrich Co, USA (product no. 17304) and Memantine hydrochloride (M) (Ebixa 10 mg tablets) was purchased from Rottendorf Pharma GmbH, Germany. GABA (SL0299Ra), Glutamate (SL1393Ra), EAAT2 (SL1690Ra) and NMDAR (QS1813Ra) Elisa kits were obtained from Sunlong Biotech Co., LTD, China.

Beta actin (Cat: E-AB-20031, RRID: AB_3662852), phosphor-pan-Akt (ser473) (Cat. no. E-AB-20802) and Goat anti-rabbit IgG (H + L) peroxidase/HRP conjugated (Cat: E-AB-1003, RRID: AB_2921220) polyclonal antibodies and super excellent chemiluminescent substrate (ECL) detection kit (Cat. E-IR-R308) were purchased from Elabscience Biotechnology Co, USA. Blue plus V protein marker (10–190 KDa) (Cat. no. DM141) and protein safe protease inhibitor cocktail (100X) (Cat. no. DI111) were purchased from Trans Gen Biotech., LTD, Peking, China. Other used materials were of the highest purity available.

### Ethical Statement

All experimental practices were authorized by the Animal Ethical Committee of the Faculty of Science, Aswan University. Ethical approval code is ASWU/05/SC/ZO/24 − 01/08.

### Animals and Experimental Designs

Fourty-eight adult male albino rats weighing 120 ± 20 g (Approximately 6–8 weeks) from the Animal House of the Egyptian Company for Vaccines in Helwan. Rats were kept in a clean, well-ventilated cage at Aswan University’s Zoology Department. They were subjected to a 12-hour light-dark cycle with a temperature of 25 ± 2 °C and a virtual humidity of 25 ± 5%. The animals were given regular food and had water *ad libtum*. Rats were allowed to adapt for one week before beginning the experiment. To avoid stress or a fight, care was taken to establish calm conditions amongst the groups being investigated. To reduce time-related differences, all treatments were given between 10:00 a.m. and 12:00 p.m. throughout the trial.

**Fig. 1:**
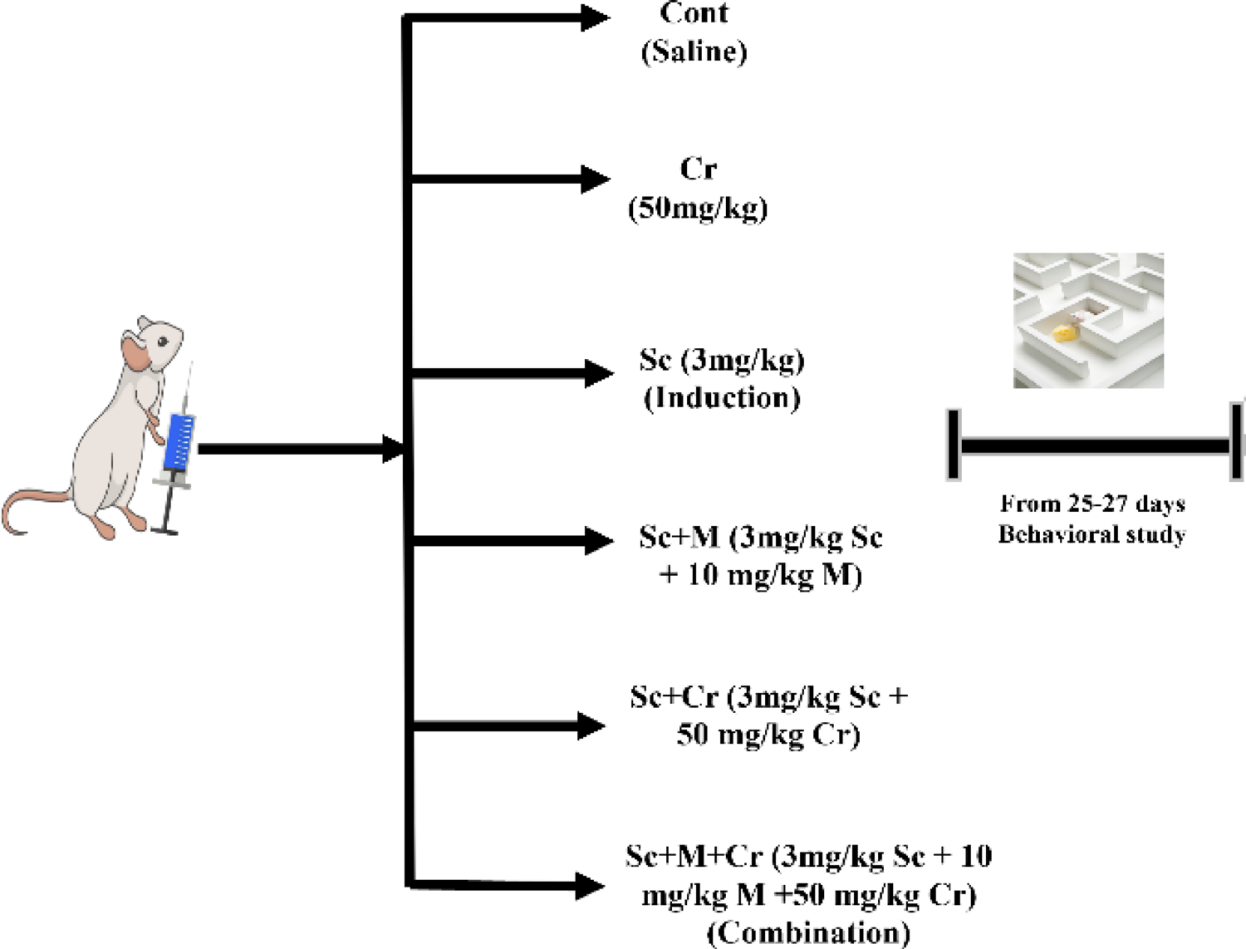
Experimental schedule of grouping, dosing, and behavioral testing timeline in the scopolamine-induced Alzheimer’s disease model in rats. Cr: crocin, Sc: scopolamine and M: memantine

The rats were categorized into six groups (*n* = 8 each) as follows (Fig. 1):

Group I - Control: Rats were treated with a daily intraperitoneal (IP) injection of saline only.

Group II: Crocin group: Designated as Cr, this group administered orally with 50 mg/kg [37] of crocin for 28 days.

Group III is the Scopolamine (induced) group. Designated as Sc, rats in this group were injected intraperitoneally with 3 mg/kg [38] of scopolamine for seven days.

Group IV: Scopolamine + memantine group: Designated as Sc + M, rats in this group were injected with 3 mg/kg of scopolamine for seven consecutive days (day 1 to day 7). Subsequently, they were treated with 10 mg/kg [39] of memantine orally for 21 consecutive days (day 8 to day 28).

Group V: Scopolamine + crocin group: Designated as Sc + Cr, rats in this group were injected with the same dose of scopolamine for seven consecutive days (day 1 to day 7). Subsequently, they were treated with crocin (50 mg/kg b. wt.) for 21 consecutive days (day 8 to day 28).

Group VI: Combination group: Designated as Sc + M + Cr, rats in this group were injected with scopolamine at the same dose for seven consecutive days (day 1 to day 7). Subsequently, they were treated with a combination of memantine (10 mg/kg b. wt.) and crocin (50 mg/kg b. wt.) for 21 consecutive days (day 8 to day 28).

#### Note

Crocin and scopolamine were freshly dissolved in saline. Similarly, memantine was freshly prepared by dissolving it in distilled water before administration.

## Behavioral Studies

### Open Field Test (OFT)

Locomotor activity and spontaneous exploratory behavior in a novel environment were assessed using the open field task, following the method described by Hall [40]. OFT was exploited to assess anxiety-like behavior of rats. Rats placed within the same corner of appurtenances; a penned square bottom (72 cm × 72 cm) surrounded by borders (36 cm high). Rats were put facing the same direction and all locomotive actions were recorded in 5 min by a video recorder camera. Measurements recorded include duration in the center area (4 squares in the middle of the apparatus), number of rearing’s, percentage of time moving, frequency of center zone, and total time spent in center zone. The floorboards were wiped with 70% ethyl alcohol between each rat.

### Novel Object Recognition Test (NORT)

In this test, animals tend to explore unfamiliar objects. It was conducted for three days—habituation, training, and testing—as described by Ennaceur and Delacour [41]. Throughout habituation, every rat was put with no objects and was permitted to explore for 5 min. Then, in the training stage, each of the rats was provided for exploring two identical objects positioned at opposing orientations. On testing day, one of the identical objects being changed with a new object (N). The apparatus was wiped with 70% ethanol following each passage. Exploration time was recorded for both familiar (F) and new objects (N). The recognition index (DI) was estimated as DI = TN/(TN + TF).

### Experimental Procedures

Animals were euthanized at the end of the experiment. Rats were anesthetized with halothane administered via inhalation, then sacrificed by decapitation. Brains dissected immediately on an ice-cold plate, and the hippocampus was isolated and divided into two parts: one for neurochemical analyses and the other for histological studies.

### Immunoblotting Techniques

#### Enzyme-Linked Immunosorbent Assay (ELISA)

Glutamate concentration, GABA concentration, GluN2B levels, EAAT2 level, and levels of p-tau were evaluated using ELISA kits, following the manufacturer’s guidelines.

#### Western Blotting

According to Maniatis et al. [42] with slight modifications, hippocampal tissues from all animals within each group (*n* = 8) were pooled to generate a representative sample. Samples homogenized in RIPA buffer containing protease and phosphatase inhibitors. The homogenate was then centrifuged at 12,000 × g for 10 min to obtain the clarified lysate for downstream protein analysis. Bradford assay was used to quantify protein concentration. A total of 100 µg of protein was denatured at 80 °C for 10 min, then separated by sodium dodecyl sulfate-polyacrylamide gel electrophoresis (SDS-PAGE). Proteins then transferred with a semi-dry transfer system onto nitrocellulose membranes. Following that, membranes were blocked using bovine serum albumin (BSA) in buffer (pH 7.4) for 1 h at room temperature. Membranes incubated at 4 °C overnight with the following primary antibodies: anti-phospho-pan Akt (Ser473; 1:1000), anti-β-actin (1:1000). After thorough washing, the membranes were incubated with a horseradish peroxidase (HRP)-conjugated secondary antibody (anti-rabbit IgG; 1:3000) for 1 h at room temperature. Bands were visualized using an enhanced chemiluminescence (ECL) detection kit, and the signals were captured. Densitometric analysis was performed using Image J software (version 6), and protein expression levels were normalized to β-actin, which served as the internal loading control.

#### Histological Examinations

The hippocampus was collected, washed with sterile saline and preserved in 10% neutral buffered formalin (pH 7). For microscopic examination, tissue samples were dehydrated in a graded ethanol series (50–99%), cleared with methyl benzoate, and embedded in molten paraffin wax at 58–62 °C. Sections were prepared at a thickness of 7 μm. These sections were then deparaffinized, rehydrated, and stained with various dyes, including hematoxylin and eosin (H&E) and Bielschowsky’s stain. H&E-stained sections were examined to assess histological changes in the hippocampus [43], while Bielschowsky’s stain was used to detect tau protein deposition [44, 45].

Histological examinations were performed using a high-power light microscope (Olympus BX43F, Tokyo, Japan). Image analysis was conducted with an Olympus DP74 digital camera and its accompanying software, connected to the optical microscope. All analyses were carried out at the Department of Zoology, Faculty of Science, Aswan University.

#### Histomorphometric and Image Analysis

A histomorphometric study was conducted to quantitatively quantify the alterations observed in the histological examinations of hippocampal tissues. Following standard histological preparation, digital images of the hippocampus were captured at 40× magnification using a digital camera mounted on a light microscope. Morphometric examination was conducted using ImageJ software (version 6), following calibration with an object micrometer to ensure accurate spatial measurements. For each animal, five representative images were selected. The following morphometric parameters were measured: thickness of the stratum pyramidal layer of CA1 of hippocampus and % tau protein immunoreactivity intensity/surface area.

#### Statistical Analysis

The data are presented as mean ± SE of at least two independent experiments. Statistical analyses (one-way ANOVA; Tukey correction; ns: not significant; *P < 0.05; **P < 0.01; ****P < 0.0001). Normality was assessed using the Shapiro-Wilk test, and all variables met the assumption of normal distribution (*p* > 0.05). Microsoft Excel and GraphPad Prism 8 were used for data processing.

## Results

### Effect of Crocin on Behavioral Studies

Based on the data presented in Table 1, the Sc-administered group indicated a significant decrease in the number of rearings (F (6, 23) = 31.19(, total movement time (F (6, 21) = 72.62), frequency to the center zone (F (6, 25) = 9.72), and cumulative time spent in the center zone (F(6, 25) = 18.27) compared to the control group (*p* < 0.0001, for all). In comparison to the Sc-administered group, the Sc + M, Sc + Cr, and Sc + M + Cr groups showed a significant increase (*p* < 0.001 and *p* < 0.0001) in these parameters (Table 1).

**Table 1:** The effect of crocin, memantine and their combination on the open field test (OFT) in various treated rats

	Cont	Cr	Sc	Sc + M	Sc + Cr	Sc + M+ Cr
NO. of rearing	23.75 ± 1.7	21.2 ± 1.46	2.2 ± 0.37****	20.75 ± 1.65***	27.75 ± 1.93***	26.5 ± 2.84***
Total time of movement (Sec)	118.48 ± 6.4	157.3 ± 7.7	34.74 ± 3.2****	157.59 ± 10.7***	179.53 ± 5.65***	220.18 ± 5.83***
Frequency of center zone	5.8 ± 0.8	5 ± 0.54	0.6 ± 0.24****	5.5 ± 0.86***	6.4 ± 0.6***	6.75 ± 1.03***
Culminative time (Sec)	6.35 ± 0.86	7.65 ± 0.3	1.9 ± 0.28****	6.54 ± 0.87**	6.56 ± 0.83**	13.32 ± 0.74***

Values are presented as means ± S.E.M of 8 animals in each group.** Highly significant compared to Sc group (p < 0.01).*** Very highly significant compared to Sc group (p < 0.001).****Very highly significant compared to control group (p < 0.0001).

There was a significant decrease in the discriminating index (F (6, 28) = 64.11) and a much shorter exploration time toward novel items (F (6, 14) = 10.77) were seen in the Sc-administered rats as compared to the control group (*p* < 0.0001, for both). However, compared to the Sc-treated group, the Sc + M, Sc + Cr, and Sc + M + Cr groups demonstrated a significant increase (*p* < 0.001) in both discrimination index and exploration duration toward the novel item (Table 2).

**Table 2:** The effect of crocin, memantine and their combination on the novel object recognition test (NORT) in various treated rats

	Cont	Cr	Sc	Sc + M	Sc + Cr	Sc + M+Cr
NORT (Sec)	37.49 ± 4.78	39.55 ± 1.35	2.56 ± 0.28^****^	36.09 ± 3.25^***^	35.74 ± 4.25^***^	39.14 ± 7.24^***^
DI %	85.58 ± 1.77	89.42 ± 1.21	39.07 ± 2.06 ^****^	77.62 ± 3.91 ^***^	81.39 ± 2.42 ^***^	84.78 ± 0.94 ^***^

Values are presented as means ± S.E.M of 8 animals in each group.**** significant compared to control group (p < 0.0001).*** significant compared to Sc group (p < 0.001).

### Effect of Crocin on Glutamate Concentration

**Fig. 2:**
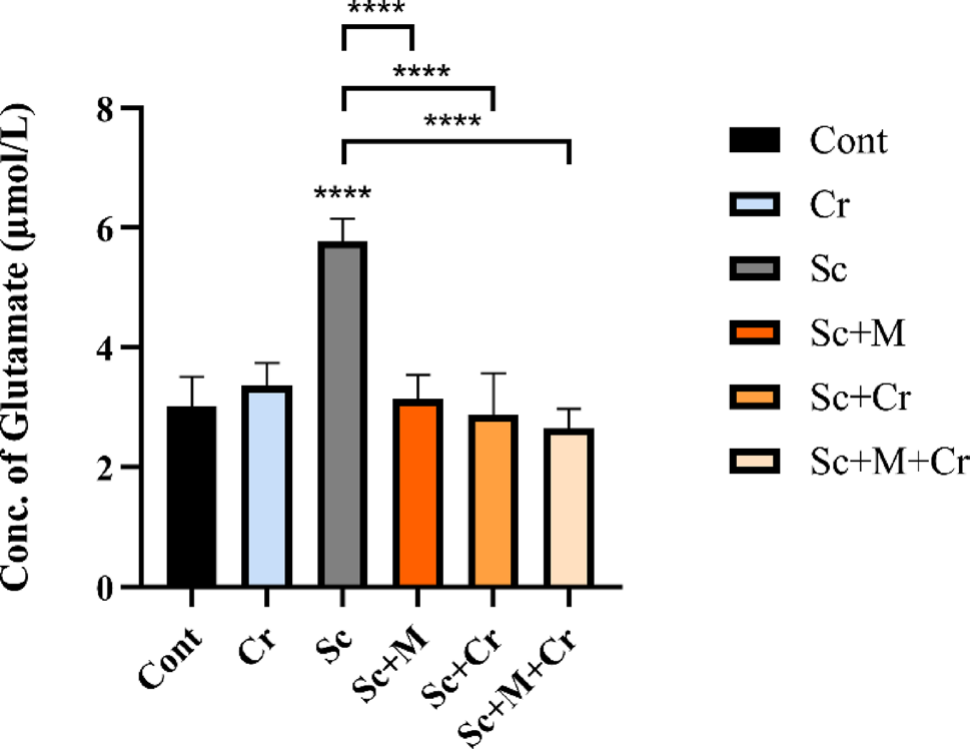
Concentration of glutamate in the brain hippocampus of adult male rats treated with scopolamine (3 mg/kg b. wt.), crocin (50 mg/kg b. wt.), memantine (20 mg/kg b. wt.) or memantine and crocin in combination. ****P < 0.0001)

In the current investigation, the Sc-administered rats presented a significant rise (F (6, 23) = 19.30, *p* < 0.0001) in hippocampus glutamate levels. The Sc + M, Sc + Cr group and Sc + M + Cr groups significantly reduced glutamate levels (*p* < 0.0001) compared to the Sc-treated group (Fig. 2).

### Effect of Crocin on GABA Concentration

**Fig. 3:**
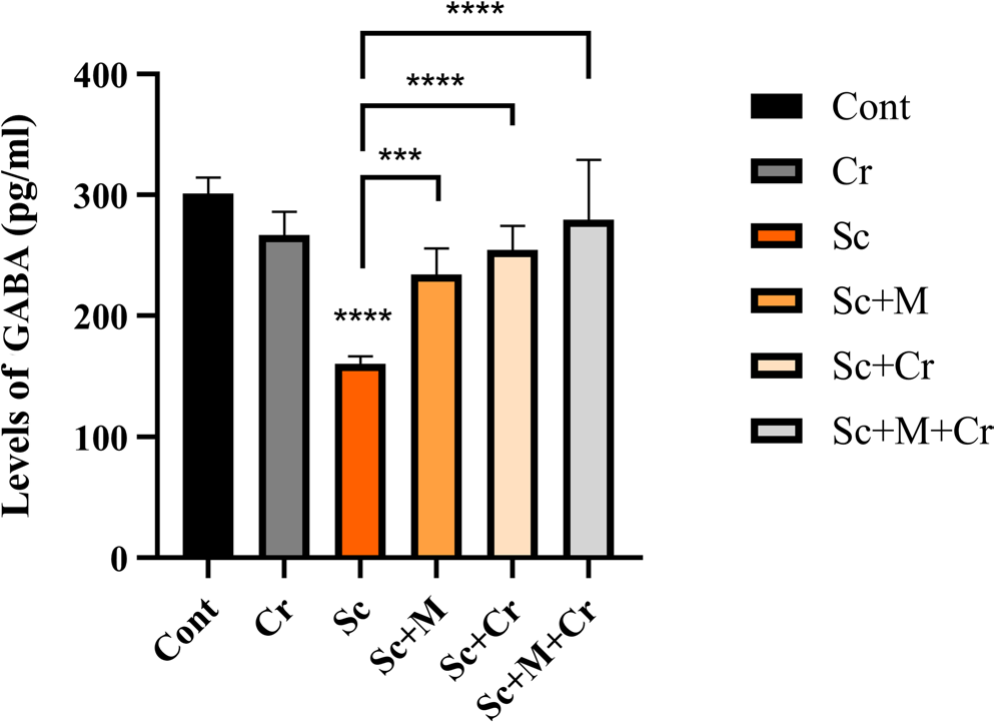
Concentration of GABA in the brain hippocampus of adult male rats treated with scopolamine (3 mg/kg b. wt.), crocin (50 mg/kg b. wt.), memantine (20 mg/kg b. wt.) or memantine and crocin in combination. ***P < 0.001; ****P < 0.0001)

The study found that Sc-administered group revealed a substantial drop (*p* < 0.0001) in GABA concentration in the hippocampus. The Sc + M group had significantly higher GABA levels (F (6, 26) = 9.47, *p* < 0.05) compared to the Sc-treated group. Also, crocin treatment, both alone (Sc + Cr group) and in combination (Sc + M + Cr group), reversed the impact of scopolamine, resulting in a significant rise (*p* < 0.001 and *p* < 0.0001) in GABA concentration compared to the Sc group (Fig. 3).

### Effect of Crocin on GluN2B

**Fig. 4:**
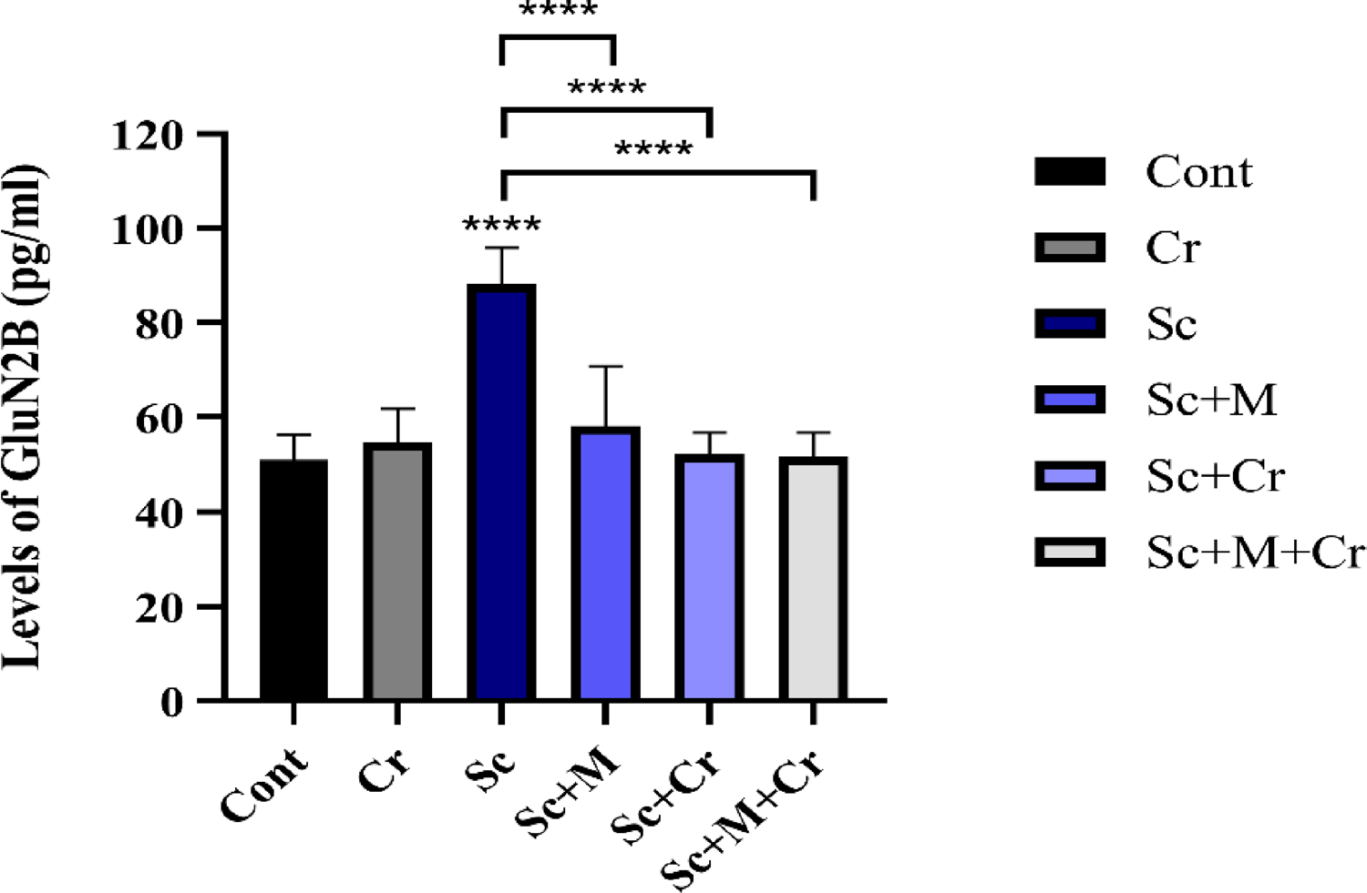
Levels of GluN2B in the brain hippocampus of adult male rats treated with scopolamine (3 mg/kg b. wt.), crocin (50 mg/kg b. wt.), memantine (20 mg/kg b. wt.) or memantine and crocin in combination. ****P < 0.0001)

As shown in Fig. 4, the Sc-treated group showed a significant elevation (F (6, 14) = 12.33, *p* < 0.0001) in the GluN2B protein levels in brain hippocampus. Conversely, treatment with memantine (Sc + M), crocin (Sc + Cr) and combination (Sc + M + Cr) groups led to a marked reduction (*p* < 0.0001) in GluN2B levels relative to the Sc group (Fig. 4).

### Effect of Crocin on EAAT2

**Fig. 5:**
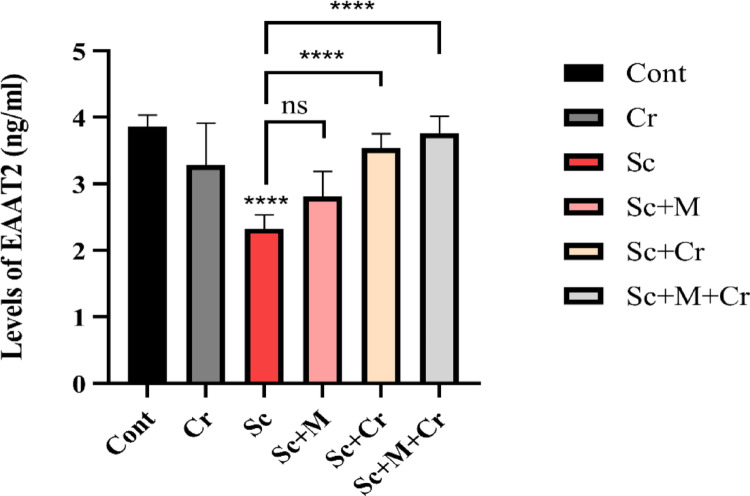
Levels of EAAT2 in the brain hippocampus of adult male rats treated with scopolamine (3 mg/kg b. wt.), crocin (50 mg/kg b. wt.), memantine (20 mg/kg b. wt.) or memantine and crocin in combination. ns: not significant; ****P < 0.0001)

This study is the first to investigate EAAT2 levels in a Sc-induced AD rat model or in response to Cr-treatment. Our findings revealed that Sc-treated group showed a substantial reduction (F (6, 14) = 26.69, *p* < 0.0001) in EAAT2 levels in the hippocampus. The Sc + M group represented a non-significant increase (*p* > 0.05) comparing to the induced group. Conversely, crocin treatment (Sc + Cr and Sc + M + Cr groups) substantially elevated EAAT2 levels (*p* < 0.0001) compared to the Sc group (Fig. 5).

### Effect of Crocin on the Expression of p-Akt

**Fig. 6:**
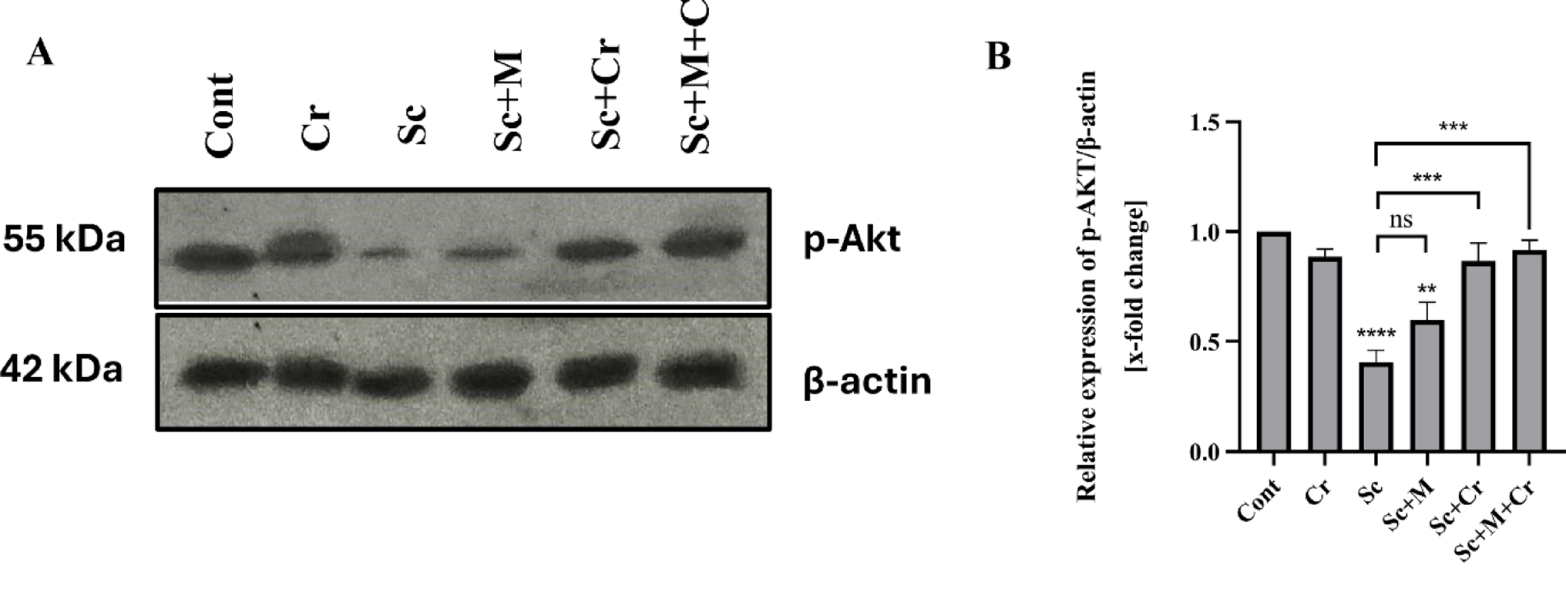
Levels of phosphorylated Akt (p-Akt) in the hippocampus of adult male rats treated with scopolamine (3 mg/kg b. wt.), crocin (50 mg/kg b. wt.), memantine (20 mg/kg b. wt.) or memantine and crocin in combination. **A** Western blot analysis showing bands for p-Akt (55 kDa) and the internal control β-actin (42 kDa). **B** X-fold change of p-Akt levels (n=3); band intensity was quantified using an image analysis system and normalized to β-actin. ns; not significant, *P < 0.05;**P < 0.01;***P < 0.001; ****P < 0.0001)

To evaluate the effect of Cr on Sc-induced alterations in protein expression, the present study employed Western blot analysis to quantify the levels of phosphorylated Akt (p-Akt) in the hippocampus. Results revealed a downregulation of p-Akt in Sc-treated rats (F (5, 12) = 16.22; *p* < 0.0001), compared to the control group. In contrast, Sc + M-treated rats showed a slight increase which was non-significant (*p* > 0.05) comparing to Sc-group. Treatment with crocin (Sc + Cr group) resulted in significant elevation of the protein expression (*p* < 0.001) when compared to Sc-group. Moreover, the combination group (Sc + M + Cr group) exhibited a notable enhancement in protein expression of p-Akt (*p* < 0.001), relative to Sc-group (Fig. 6).

### Effect of Crocin on Phosphorylated Tau (p-tau) Levels

**Fig. 7:**
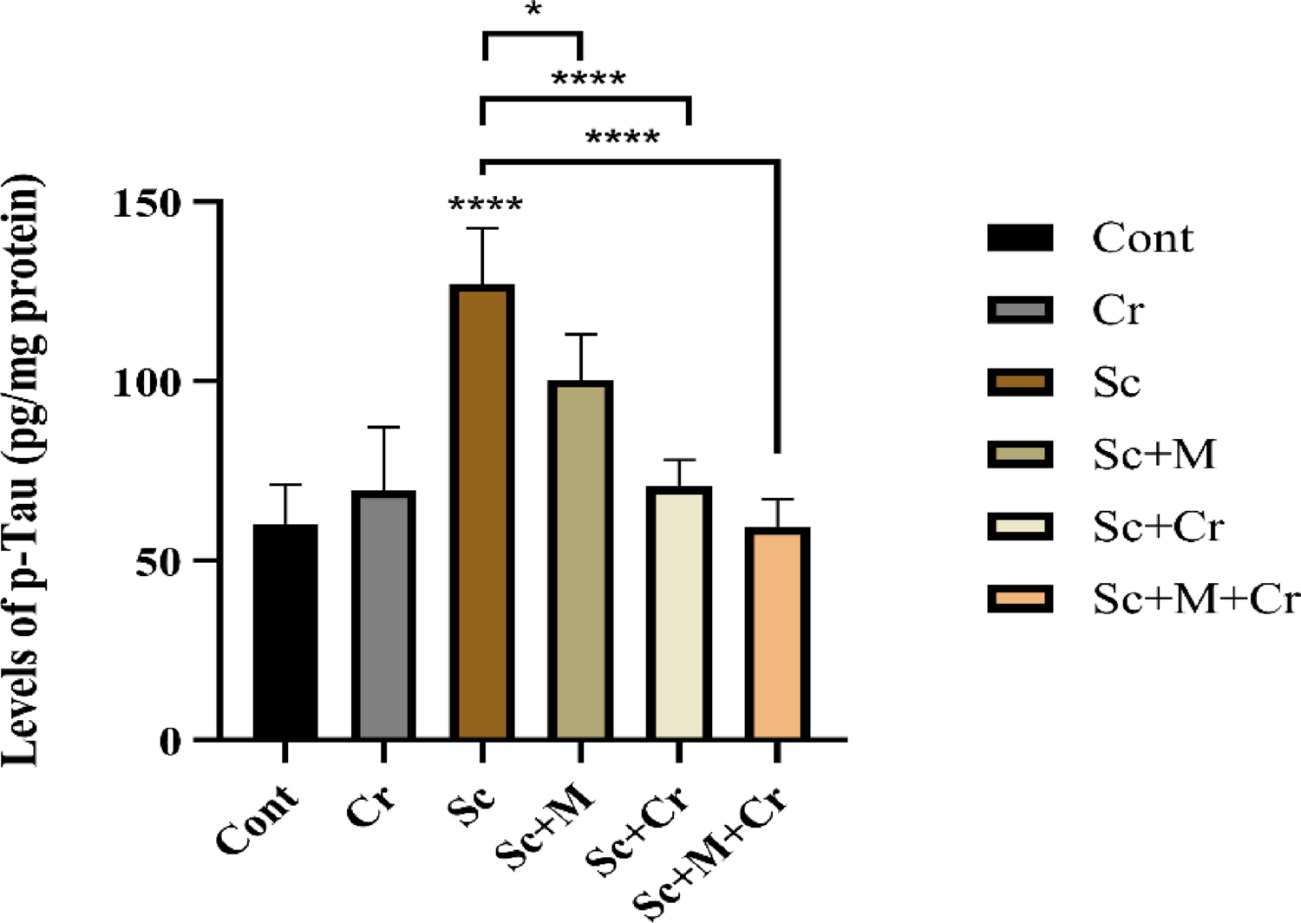
Levels of p-Tau in the brain hippocampus of adult male rats treated with scopolamine (3 mg/kg b. wt.), crocin (50 mg/kg b. wt.), memantine (20 mg/kg b. wt.) or memantine and crocin in combination. *P < 0.05; ****P < 0.0001

Our findings demonstrated that the p-tau protein level in the hippocampus of Sc-administered rats was considerably higher (F (6, 14) = 28.53, *p* < 0.0001) compared to the control group. Conversely, the Sc + M group showed a significant decrease in p-tau levels (*p* < 0.05) compared to the Sc group. Moreover, a highly considerable drop (*p* < 0.0001) in p-tau levels was observed in the hippocampus of the Sc + Cr and Sc + M + Cr groups when compared to the Sc-treated rats (Fig. 7).

### Effect of Crocin on the Histological Structure of the Hippocampus

**Fig. 8:**
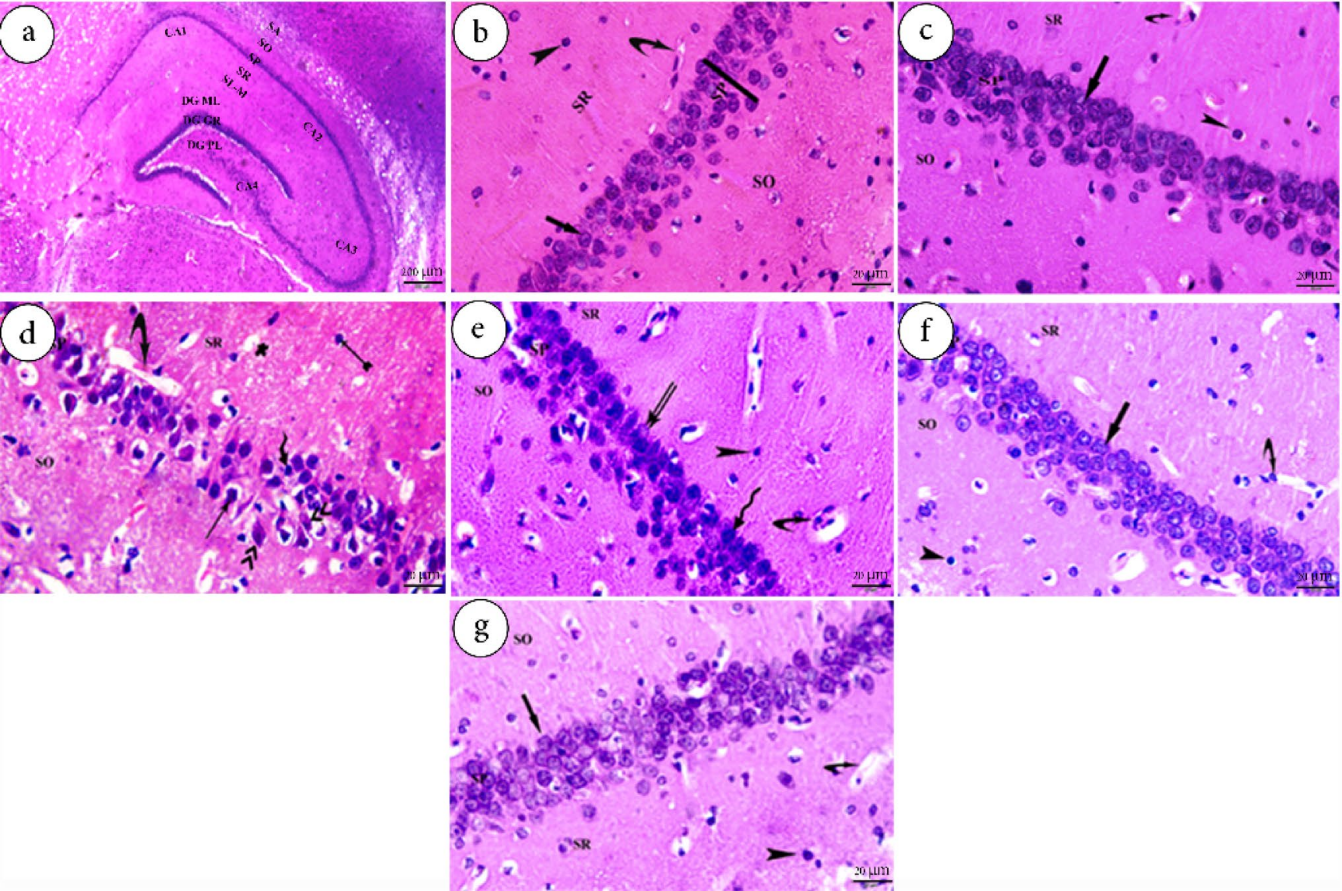
Photomicrographs of the hippocampus of adult male albino rats stained with H&E (bar = 20 μm). **a** and **b** show sections from the control group; **c** shows the Cr-treated group; **d** shows the Sc group; **e** shows the Sc + M group; **f** shows the Sc + Cr group; and **g** shows the combination group (Sc + M + Cr group). Anatomical regions include Cornu Ammonis (CA), dentate gyrus (DG), stratum alveus (SA), stratum oriens (SO), stratum pyramidale (SP), stratum radiatum (SR), and stratum lacunosum-moleculare (SL-M). Histological features include pyramidal neurons with vesicular nuclei and visible nucleoli (thick arrows), glial cells (arrowhead), blood capillaries (curved arrow), intense eosinophilia (thin arrow), and nuclear basophilia (zigzag arrow). Additional pathological signs include fluid accumulation and vacuolation (star), pyknotic nuclei of glial cells (forked arrows), and karyorrhectic nuclei (double arrowheads). H&E: Hematoxylin and Eosin; Cr: Crocin; Sc: Scopolamine; M: Memantine

Microscopic examination of H&E-stained sections of the hippocampus in the control group revealed a normal hippocampal structure, including the Cornu Ammonis (CA) and dentate gyrus. The CA is subdivided into four distinct regions: CA1, CA2, CA3, and CA4. Each of these regions consisted of five layers arranged from outer to inner; include the stratum alveolus (SA), stratum oriens (SO), stratum pyramidale (SP), stratum radiatum (SR), and stratum lacunosum-moleculare (SL-M) (Fig. 8a). In the CA1 region, the SP of both the control and Cr-treated groups consisted of 4–5 compact layers of large pyramidal neurons with vesicular nuclei and prominent nucleoli (Fig. 8b and c, respectively).

A photomicrograph from the Sc-induced group revealed numerous histopathological alterations in the CA1 region of the hippocampus. These changes included a decreased thickness of the SP cell layer, which is a widely accepted indicator of neuronal necrosis. Additionally, vascular injury was observed, characterized by fluid accumulation in the neuropil, resulting in a vacuolated appearance. Numerous necrotic, condensed (pyknotic) nuclei of glial cells were also evident (Fig. 8 d) in both SR and SO regions.

Sections from the Sc + M-treated group showed loosely arranged layers of the SP with signs of neuronal necrosis. The SO and SR also exhibited necrotic, condensed nuclei of glial cells, along with congested blood capillaries (Fig. 8e). In contrast, sections from the crocin-treated group (Sc + Cr) and the combination group (Sc + M+Cr) demonstrated preserved structural integrity of the hippocampus. The CA1 region displayed a well-organized SP with compact layers of large pyramidal neurons, most of which had vesicular nuclei and prominent nucleoli. Blood capillaries and glial cells appeared normal in both SR and SO regions (Fig. 8f & g, respectively).

**Fig. 9:**
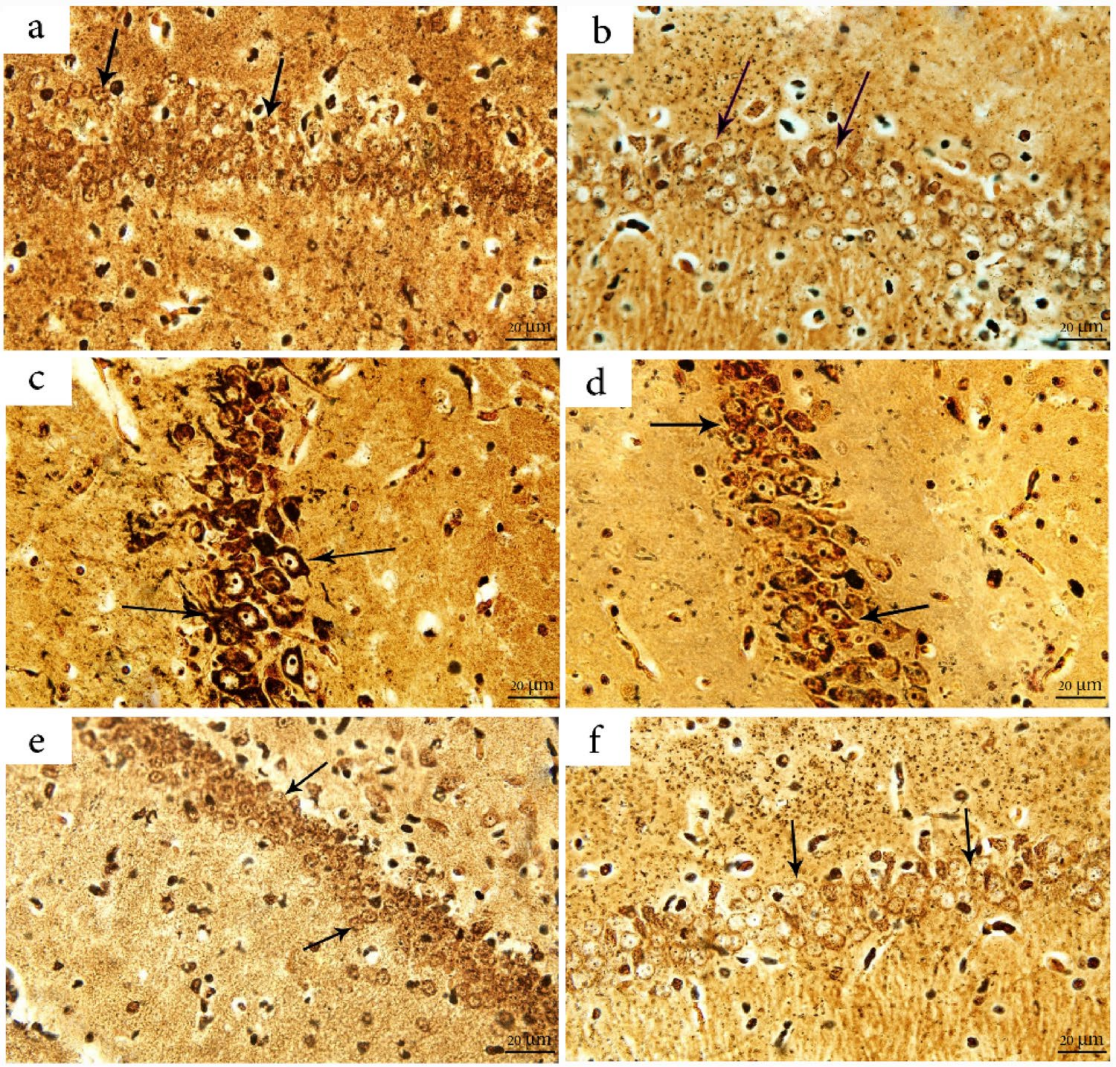
Photomicrographs of the hippocampus of adult male albino rats stained with Bielschowsky’s silver stain (bar = 20 μm). **a** shows a section from the control group; **b** shows the Cr-treated group; **c** shows the Sc group; **d** shows the Sc + M group; **e** shows the Sc + Cr group; and **f** shows the combination group (Sc + M + Cr) group. Arrows indicate the region of interest; intense tau protein deposition is observed in SC-treated groups compared to controls. Cr: Crocin; Sc: Scopolamine; M: Memantine

Examination using the Bielschowsky silver staining technique revealed normal neuronal morphology in the CA1 region of both the control and Cr-treated groups, with neurons exhibiting the characteristic dark yellow to brown staining against a yellow background (Fig. 9a & b, respectively). In contrast, hippocampal sections from the Sc-treated group showed intense dark brown staining in the CA1 neurons, indicative of substantial tau protein deposition (Fig. 9c). Additionally, the Sc + M group displayed moderate brown staining intensity, suggesting a mild level of tau accumulation in the SP of the CA1 region (Fig. 9 d). Sections from the crocin-treated groups, including the Sc + Cr group and the Sc + M+Cr combination group, showed markedly reduced staining intensity, with faint brown coloration indicating a near-normal distribution of tau tangles (Fig. 9e & f, respectively).

### Morphometric Assessments

#### Thickness of the Stratum Pyramidale (SP)

Morphometric analysis of hematoxylin and eosin (H&E)-stained hippocampal sections revealed a highly significant reduction (F (6, 14) = 16.72, *P* ≤ 0.001) in the thickness of the stratum pyramidale (SP) within the CA1 region in the Sc-treated group compared to both the control and Cr-treated groups. In contrast, the Sc + M group showed a statistically significant increase (*P* < 0.05) in SP thickness relative to the Sc-treated group. Furthermore, treatment with crocin, either alone (Sc + Cr group) or in combination (Sc + M+Cr group) resulted in a highly significant increase (*P* ≤ 0.0001) in SP thickness compared to the Sc-treated group (Table 3).

**Table 3:** The effect of crocin, memantine and their combination on the thickness of CA1 layers and intensity of Tau protein deposits of brain hippocampus in various treated rats

	Cont	Cr	Sc	Sc + M	Sc + Cr	Sc + M+Cr
Thickness of CA1 layer (µm)	39.33 ± 2.03	44 ± 3.21	25 ± 2.08^***^	29.66 ± 0.88^*^	42.66 ± 1.2^****^	41.33 ± 0.88^****^
% of tau protein intensity	12.27 ± 0.964	10.65 ± 0.506	26.37 ± 0.977 ^***^	22.32 ± 1.17 ^*^	14.77 ± 0.901 ^****^	11.15 ± 1.27 ^****^

Values are presented as means ± S.E.M. of 5 animals in each group.

* Significant compared with Sc group (p<0.05).

***Highly significant compared with Sc group (p<0.001).

**** Highly significant compared with control group (p<0.0001).

#### Intensity of Tau Protein Depositions in the CA1 Region of the Hippocampus

Consistent with the observed alterations in SP layer thickness, histochemical morphometric analysis revealed a markedly increased tau protein deposition in the hippocampus of Sc-treated rats, as indicated by significantly intensified staining (F (6, 21) = 35.71, *P* < 0.001) compared to the control. In contrast, the Sc + M group showed a significant decrease (*P* < 0.05) in tau deposition relative to the Sc-treated group. Moreover, a highly significant reduction (*P* ≤ 0.0001) in tau staining intensity was observed in the crocin-treated groups, both alone (Sc + Cr) and in combination (Sc + M + Cr), compared to the Sc-treated group (Table 3).

## Discussion

Alzheimer’s disease is a sort of dementia that is distinguished by impaired cognition, reduced memory, and behavioral changes. In this study, we used intraperitoneal (IP) injection of scopolamine to simulate an aging-related model of Alzheimer’s disease. A combined treatment with scopolamine and crocin was explored as a potential approach for developing novel therapeutic agents for the disease. Scopolamine, a muscarinic receptor antagonist, is considered to be the gold standard to induce cognitive issues in healthy individuals and animals, as it mimics the characteristic muscarinic dysfunction observed in dementia [46].

Cognition, a neurological process of understanding, such as knowledge, thinking, interpretation, and decision-making [47]. Accordingly, under our experimental conditions, the impacts of scopolamine on learning and memory were investigated using both the open field and novel object recognition tasks. The open field test was applied to evaluate behavioral exploration and locomotor activity in rats exposed to a novel environment [48]. Rats treated with scopolamine exhibited reduced crossing and rearing behaviors, indicating altered locomotor activity due to scopolamine. The novel object recognition test (NORT) is a widely used method to assess short-, intermediate-, and long-term memory alterations. Animals naturally tend to explore novel objects due to their innate curiosity. In the present study, 3 mg/kg of scopolamine induced deficits in memory recognition, as evidenced by reduced object discrimination and a lower discrimination index (%).

These results correspond to those of Ishola et al. [49], Yadang et al. [50], Chaturvedi et al. [51], Kantar et al. [52], and Thongrong et al. [53], who demonstrated that animals treated with scopolamine exhibited significant impairments in spatial and non-spatial learning, memory, and overall cognitive function. Scopolamine treatment resulted in a reduced number of crossings, rearing behaviors, and time spent in the center of the apparatus, indicating altered locomotor activity and a significant decline in the discrimination index. Moreover, other studies have demonstrated that scopolamine-treated rats showed reduced exploratory behavior and a marked decrease in the investigation of novel objects [54, 55].

Evidence indicates that glutamate binds to specific receptors (NMDA-R) located on postsynaptic neurons. These receptors stimulate sodium and calcium ions influx into the neurons [56–59]. To prevent glutamate receptors hyperactivation, excessive synaptic glutamate must be rapidly removed. EAAT2 is responsible for the uptake of approximately 80–90% of extracellular glutamate [60]. Additionally, dysfunction of the GABAergic system is observed in the early stages of the disease and is influenced by a difference between excitation and inhibition [61–64].

This study observed that scopolamine administration led to elevated glutamate levels and increased GluN2B protein levels, while GABA concentrations and EAAT2 levels were significantly reduced. Our findings are supported by the studies of Garabadu and Sharma [65], and Asadi Rizi et al. [66], who also reported increased glutamate levels and enhanced NMDA receptor activity in scopolamine-treated rats. Scopolamine promotes the upregulation of certain synaptic proteins, such as GluN2B, and may increase the number and function of NMDA receptor-associated synapses. Previous results also found that prolonged activation of NMDA receptors leads to extreme glutamate release, which in turn causes neuronal damage and cell death [67–70]. In the same context, similar observations were stated by Deng et al. [71] and Oyetayo et al. [72], indicating that scopolamine-induced dementia was accompanied by decreased GABA levels and increased glutamate levels. These results are in line with earlier studies [73–76].

Based on the available literature, the levels of EAAT2 have not been previously estimated in a scopolamine model of Alzheimer’s disease (AD). While scopolamine is traditionally used to induce cholinergic deficits and cognitive impairment, emerging evidence suggests that it may also promote the accumulation of amyloid-beta (Aβ) oligomers, thereby extending its relevance to both glutamatergic and amyloid-related mechanisms in AD pathology [77, 78]. In particular, Aβ oligomers have been shown to disrupt EAAT2 function by reducing its membrane expression in astrocytes [79, 80], leading to impaired glutamate clearance and elevated glutamate concentration. This dysfunction results in excessive activation of NMDA receptors, ultimately contributing to synaptic failure and neurodegeneration [80, 81]. Interestingly, while EAAT2 is often downregulated in the hippocampus of AD models, recent findings by Wood et al. [19] suggest that its expression may be upregulated in other brain regions such as the prefrontal cortex. This regional variability may reflect differences in astrocytic density, local glutamatergic activity, and region-specific regulatory mechanisms of EAAT2.

The PI3K/Akt signaling axis is one of the most crucial pathways for neuronal survival. Glycogen synthase kinase-3β (GSK-3β), a downstream target of the PI3K/Akt pathway, becomes active and promotes the phosphorylation of tau protein at various sites [82]. In the current investigation, scopolamine decreased the phosphorylation levels of Akt, reducing its activity. This, in turn, increased the levels of phosphorylated tau protein. These findings match Zhao et al. [83], who reported that a 3 mg/kg dose of scopolamine reduced the expression of p-Akt. Scopolamine-induced memory deficits are associated with Akt inactivation and GSK-3β activation [84].

Similarly, Mostafa et al. [85] and Magadmi et al. [86] indicated that scopolamine administration give rise to a decline of phosphorylated Akt (p-Akt), a key component of neuronal survival pathways. Recent studies by Yang et al. [82], Xiong et al. [87], Razani et al. [88], and Farhat et al. [23] suggested that reduced phosphorylation of Akt at the Ser473 residue, along with decreased phosphorylation of GSK-3β at the Ser9 site, leads to hyperphosphorylation of tau protein, thereby accelerating its accumulation and fibrillation.

The observed changes in glutamate levels, overstimulation of NMDA receptors and subsequent excitotoxicity appear to trigger an inhibition of the Akt, as evidenced by reduced phosphorylation at Ser473 [23]. In turn, diminished Akt activity permits the activation of GSK-3β, a kinase concerned with the pathological hyperphosphorylation of tau protein. These alterations may be attributed, at least in part, to altered EAAT2 levels. Our findings suggest that EAAT2 downregulation could act as a trigger for excitotoxicity and neurodegenerative processes in this model.

The hippocampus is highly susceptible to damage, particularly in relation to learning and memory functions. It is among the earliest brain regions affected by neurodegeneration in Alzheimer’s disease, making the assessment of structural and functional changes in this area crucial for AD diagnosis. In the present study, the induced group displayed numerous histopathological alterations throughout most areas of the CA1 region of the hippocampus. These alterations included a decreased thickness of the pyramidal cell layer. The most widely accepted histological evidence of neuronal necrosis includes cytoplasmic shrinkage and intense eosinophilia, accompanied by nuclear shrinkage and increased basophilia. Additionally, vacuolation was observed, along with the presence of necrotic, condensed (pyknotic) nuclei in glial cells. In agreement with these findings, Abd-El-Fattah et al. [89], Safar et al. [90], Barai et al. [91], and Sameni et al. [92] established that scopolamine significantly increased the number of dark cells, neuronal shrinkage, vacuole formation, glial activation, and indistinct cellular membranes in the CA1 region of the hippocampus compared to the control group.

In the present study, crocin exhibited significant neuroprotective effects in a scopolamine-induced Alzheimer’s disease (AD) rat model, as evidenced by behavioral, neurochemical, and histological parameters. The protective, curative, and combination groups treated with crocin showed improved cognitive function, as assessed by the open field test (OFT) and novel object recognition test (NORT). These improvements are likely attributed to crocin’s well-documented neuroprotective properties against hippocampal damage. Several previous studies support these findings; for instance, Singh [93] highlighted crocin’s cognition-enhancing effects on both spatial and recognition memory. Similarly, Hadipour et al. [94] reported that crocin counteracts amyloid-beta-triggers learning and memory deficits, while Mohammadzadeh et al. [95] found that crocin pretreatment reduced malathion-induced impairments. Ghofrani et al. [96] also confirmed crocin’s protective role against trimethyl tin (TMT)-induced cognitive decline, attributing its effects to its antioxidative properties.

Consistent with these behavioral outcomes, crocin demonstrated significant biochemical modulation of the glutamatergic and GABAergic systems. In this study, crocin treatment resulted in decreased glutamate levels and NMDA receptor activity, along with increased GABA concentration in the hippocampus. These changes suggest that crocin mitigates glutamate excitotoxicity, one of the central contributors to neurodegeneration in Alzheimer’s disease (AD). Glutamate excitotoxicity is a key pathogenic mechanism in neurodegenerative disorders, and reducing glutamate release or enhancing its clearance has been shown to be an effective therapeutic strategy [97–99]. Supporting our findings, Yousefvand et al. [100] demonstrated that crocin can modulate NMDA receptor activity by antagonizing ketamine’s binding. Similarly, Finley and Gao [67] showed that crocin reversed ketamine-induced memory deficits. Moreover, the roles of NMDA and AMPA receptors in synaptic plasticity and memory formation are well established [101, 102], and crocin has been shown to positively influence these pathways [103, 104]. Also, Hassani et al. [105] demonstrated that crocin alleviated hyoscine-induced memory impairments, consistent with our observations.

As we mentioned before, our study is the first to investigate EAAT2 in a model of scopolamine-induced AD and treated with crocin. The current results presented a highly significant increase in the levels of EAAT2 after administration of crocin whether alone or in combination. This could be due to the anti-amyloidogenic and antioxidant effect of crocin [106, 107].

At the molecular level, the neuroprotective mechanisms of crocin involve regulation of the PI3K/Akt/GSK3β signaling pathway. Our data show that crocin activated Akt, which in turn inhibited GSK3β activity, leading to reduced tau hyperphosphorylation and potentially limiting neurofibrillary tangle formation. This protective mechanism is supported by Yang et al. [82], Mohammadzadeh et al. [95], Sadoughi [108], and Salama et al. [109], who reported an increase in the levels of phosphorylated Akt, along with reduced levels of phosphorylated tau following crocin administration. Crocin’s antioxidant properties may also contribute to this effect, as suggested by Ahmed et al. [110], who reported improved spatial cognition and reduced tau protein levels. Previous studies have shown that activation of the PI3K/Akt pathway can upregulate EAAT2 expression in astrocytes [111], providing a mechanistic basis for the observed increase in EAAT2 levels following crocin treatment. This suggests that crocin may exert its neuroprotective effects, at least in part, through Akt-mediated regulation of glutamate transport. Collectively, these findings underscore crocin’s potential to protect neurons from tau-related pathology through modulation of this critical intracellular pathway.

In addition to these intracellular mechanisms, the pharmacokinetic properties of crocin further support its central neuroprotective potential. Although crocin itself exhibits limited oral bioavailability due to poor absorption, it is metabolized into crocetin—a lipophilic derivative capable of crossing the blood-brain barrier (BBB) and exerting central effects [112, 113]. Crocetin has been detected in brain tissue following systemic administration, suggesting that crocin’s therapeutic effects may be mediated through its active metabolite [114]. Additionally, crocin has been shown to preserve BBB integrity under pathological conditions, such as ischemia and neuroinflammation, thereby preventing secondary neuronal damage [115]. Mechanistically, crocin and crocetin exert antioxidant, anti-inflammatory, and anti-apoptotic effects by modulating key signaling pathways, including PI3K/Akt and MAPK [116, 117]. These properties contribute to their ability to counteract excitotoxicity, reduce tau hyperphosphorylation, and enhance synaptic plasticity.

Histological analysis further confirmed crocin’s neuroprotective effects. In the treated groups, the hippocampal CA1 region displayed preserved structural integrity, with a well-organized stratum pyramidale and healthy pyramidal cells. Blood capillaries and glial cells also appeared normal. These results align with Baghishani et al. [118] who stated that crocin lowered apoptotic cells and the number of dark neurons in the CA1 region. Hadipour et al. [119, 120] further demonstrated crocin’s protective role against β-amyloid-induced neurotoxicity in the CA1 region. Sameni et al. [92] observed that crocin minimized scopolamine-induced damage in the CA1 region by decreasing the number of dark neurons and increasing the population of viable cells. Additionally, Krishnaswamy et al. [121] reported that crocin administration decreased the proportion of apoptotic cells and preserved tissue morphology in the cerebral cortex of neurodegenerative models.

Combination therapy with crocin and memantine yielded additive effects, which may be attributed to their complementary mechanisms. While memantine reduces NMDA receptor overactivation, crocin acts upstream by restoring EAAT2-mediated glutamate clearance and downstream by modulating tau-related pathways. This multitarget approach mirrors the growing consensus that effective AD therapy requires modulation of multiple pathological cascades rather than single targets.

## Conclusion

In conclusion, our study demonstrates that crocin not only ameliorates scopolamine-induced neurotoxicity but also provides novel evidence for EAAT2 levels upregulation as a central mechanism of action. These findings, supported by previous literature, highlight crocin—either alone or combined with memantine—as a promising multitarget therapeutic candidate for AD. A last, a limitation of this study is that only male rats were used. Including female rats in future experiments would help to reveal possible sex-related differences and make the findings more generalizable.

## Supplementary Information

Below is the link to the electronic supplementary material.

Supplementary Material 1

## Data Availability

This article contains all the data that was created or evaluated during the research.
