# Supplementary figures and images for: Crocin Mitigates Glutamate Excitotoxicity and Tau Hyperphosphorylation by Modulating EAAT2 and Akt/Tau Pathway in a Scopolamine-induced Rat Model of Alzheimer’s Disease
Source: Neurochem Res. 2026 Mar 5;51(2):100. doi: 10.1007/s11064-026-04692-z (PMC12963091; doi:10.1007/s11064-026-04692-z)

**Original blot of p-Akt and β-actin**

**
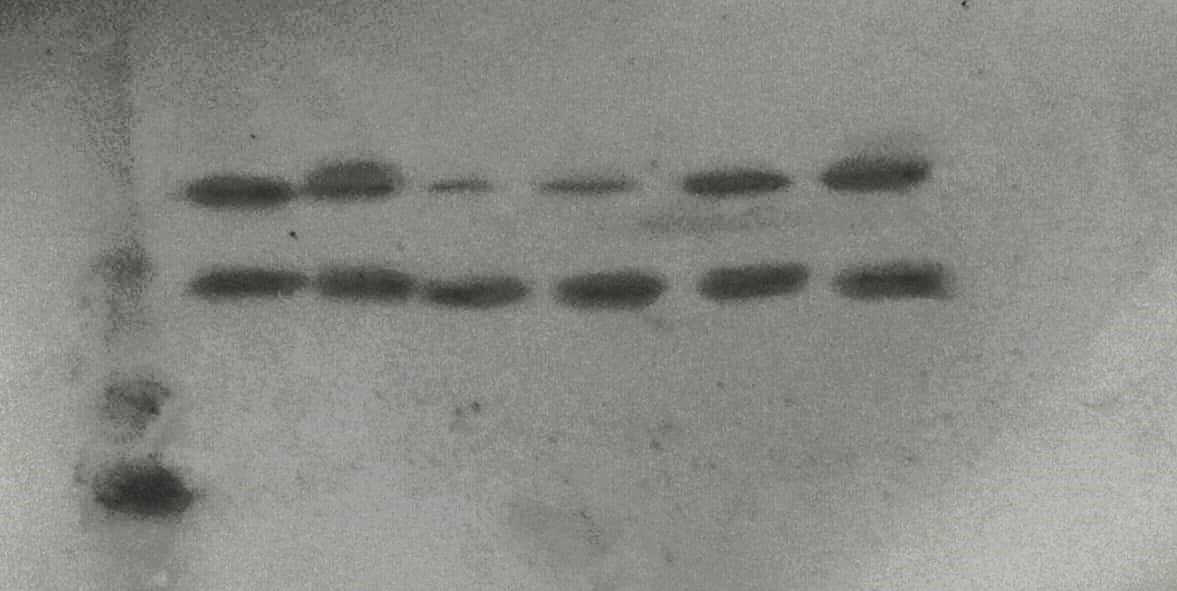
**

**Quantifications**

| **Groups** | **p-Akt** | **Β-actin** |
| --- | --- | --- |
| Cont | 22371.522 | 22777.229 |
| Cr | 21437.936 | 26265.149 |
| Sc | 4979.368 | 21299.300 |
| Sc+M | 8534.338 | 22415.057 |
| Sc+Cr | 16879.936 | 22310.108 |
| Sc+M+Cr | 19666.693 | 23105.714 |

Supplement: Supplementary file 1 — Supplementary Material 1 [file 11064_2026_4692_MOESM1_ESM.docx]
